# Perceived control, loneliness, early-life stress, and parents’ perceptions of stress

**DOI:** 10.1038/s41598-023-39572-x

**Published:** 2023-08-10

**Authors:** Karen E. Smith, Eileen Graf, Kelly E. Faig, Stephanie J. Dimitroff, Frederica Rockwood, Marc W. Hernandez, Greg J. Norman

**Affiliations:** 1 University of Chicago, Chicago, Illinois 60637 USA; 2 Present Address: Rutgers University-Newark, Smith Hall Rm 341, 101 Warren St, Newark, NJ 07102 USA; 3 NORC at the University of Chicago, Chicago, Illinois USA

**Keywords:** Human behaviour, Social neuroscience, Stress and resilience

## Abstract

The COVID-19 pandemic has highlighted the importance of understanding what contributes to individual variability in experiences of stress. Increases in stress related to the pandemic have been especially pronounced in parents, indicating a need for research examining what factors contribute to parents’ perceptions of stress. Here, we assessed the relationship between parents’ perceptions of stress, control, loneliness, and experiences of childhood trauma in two populations of caregivers. In Study 1, we examined the relationship between perceptions of stress, control, loneliness, and history of early stress, along with indices of socioeconomic risk and resting parasympathetic nervous systema activity, which has been linked to variability in perceptions of stress, in caregivers of young children. Perceived control, loneliness, childhood stress, and resting parasympathetic nervous system activity predicted caregivers’ stress. In Study 2, we replicated these initial findings in a second sample of caregivers. Additionally, we examined how these processes change over time. Caregivers demonstrated significant changes in perceptions of control, loneliness, and stress, and changes in control and childhood trauma history were associated with changes in perceptions of stress. Together these results indicate the importance of assessing how caregivers perceive their environment when examining what contributes to increased risk for stress. Additionally, they suggest that caregivers’ stress-related processes are malleable and provide insight into potential targets for interventions aimed at reducing parents’ stress.

## Introduction

The outbreak of the global pandemic associated with COVID-19 has brought to the forefront the question of how to ameliorate chronic or extreme levels of stress. Increases in perceived stress during the pandemic have been especially pronounced in parents^1–3^, highlighting a need to better understand what factors increase (or decrease) a parents’ risk for chronic or extreme levels of stress. Becoming a parent already represents an important source of potential stress, constituting a major event associated with significant life changes that affect priorities, routines, and relationships. While this stress in and of itself is not necessarily harmful (indeed, it likely plays an important role in motivating effective parenting behaviors), chronic and/or extreme stress associated with parenting has been demonstrated to negatively impact parenting behaviors^4,5^ and, subsequently, child outcomes^6,7^. Better understanding how factors implicated in stress perceptions, including perceptions of control, loneliness, prior exposure to stress in childhood, and physiological indices linked to stress perception influence parents’ perceptions of stress can aid in informing targeted interventions for parents at risk for high levels of stress.

Stress can refer broadly to demands placed upon an individual requiring adaptation or change. The demands are referred to as stressors and conceptualized as a potential threat or challenge to the individual’s equilibrium^8,9^. Stress responses, in turn, support motivated physiological, psychological, and behavioral changes aimed at addressing the potential threat or challenge^10^. Predominant models of stress posit that an individual’s perception of something as a potential threat, real or imagined, rather than the event itself, drive a significant proportion of stress reactivity and its corresponding psychological, physiological, and behavioral consequences^9,11,12^ (but see Bonanno^13^ and McLaughlin et al.^14^ for alternative perspectives). While stress responses are typically beneficial, motivating individuals to respond effectively to their environment^8^, chronic stress has the potential to result in dysregulation of these responses via extended activation and disequilibrium of stress systems. This dysregulation has been linked to increased mental and physical health risk^15,16^. Indeed, disequilibrium and associated perceptual and cognitive shifts have been posited to be characteristic of stress disorders, including acute stress disorder and post-traumatic stress disorder (PTSD)^17–19^. In parents, it is chronic and/or extreme stress that is associated with negative parenting behaviors and poorer child outcomes^20^.

Whether an individual perceives a given event as a stressor is multiply determined by a number of environmental, individual, and biological factors. Theoretical frameworks of stress have proposed that key moderating factors in how threats are perceived and coped with include perceptions of control over the stressor, perceptions of the quality of social relationships, and parent exposure to childhood trauma^9–11^. Perceived control, which refers to believing one has adequate resources available to respond to and manage a potential stressor^21^, has been linked to reduced perceived stress and dampened physiological and neural responses to acute laboratory stress^22–27^. Similar findings have also been demonstrated for individuals who report having a high internal locus of control—people who perceive outcomes to be decided by their own actions rather than chance^23–25^. Across these studies, individual responses varied despite being exposed to the same type, intensity, and duration of stimulus (i.e., the same amount of painful stimulation for the same amount of time), demonstrating that perceptions of control can dramatically change both perceptual, physiological, and neural responses to the same physical stimulus (e.g. a shock).

Perceiving oneself to lack high quality and meaningful social relationships, referred to as loneliness^28,29^ is also linked to altered experiences of stress. Loneliness arises from an individual’s perceptions of isolation and loss of social connection and does not necessarily correlate with the actual number of relationships they have^28^. Recent models of loneliness describe it as a negative affective state that represents a psychological stress response to the social threat of lacking social relationships^30^. In this perspective, the perceived loss of social connections central to loneliness results in a coordinated set of psychological, physiological, and behavioral responses aimed at addressing this perceived threat. In particular, loneliness is thought to facilitate increased vigilance towards social threats. Indeed, loneliness has been linked to cognitive biases towards negative social information, including increased attention toward socially threatening stimuli and increased sensitivity to negative emotional states in others^31–33^. In this way, loneliness can be characterized as a stress response. But, this resulting hypervigilance to social threat also has the potential to further exacerbate perceptions of stress in general, increasing the likelihood of individuals perceiving events and social interactions as threatening^11,28^. In support of this, loneliness is associated with both increased perceptions of stress as well as increased sensitivity to threat in the environment^34,35^.

Previous experiences with stress additionally influence current stress by shifting how individuals perceive and interpret their environment. Chronic and/or extreme stress occurring in childhood, referred to as early life stress, can have an especially pronounced effect^15,36^. A history of early childhood trauma is associated with hypersensitivity to threat in the environment and increased risk of continued chronic perceptions of stress thought to lead to a marked disruption of stress response systems^15,37–40^. Researchers have proposed that these shifts may result in individuals exposed to severe early life stress being more prone to perceiving neutral or ambiguous events within their environment as threatening^39–41^. In support of this, early childhood abuse and trauma is related to increased sensitivity to threat, particularly in the context of ambiguity^16,42,43^, and increased levels of perceived stress in adulthood^44–46^.

Last, research suggests that variability in biological factors, like parasympathetic autonomic cardiac functioning, are also associated with increased sensitivity to threat^47,48^. The parasympathetic nervous system (PNS) is one branch of the autonomic nervous system (ANS) which is critical in coordinating adaptive responses to stress^49–51^. High resting PNS activity has been linked to differences in sensitivity to threat and safety cues in the environment^48,52,53^, reduced negative affect in response to stress^54^, and lower levels of perceived stress in adults. Resting PNS has also been found to facilitate differential responses to loneliness^55^ and decreased risk for psychopathology after experiences of childhood adversity^56,57^, suggesting it may moderate relationships between factors like loneliness, control, early experiences, and stress. Together this research indicates that perceptions of stress are multiply influenced by a number of complex processes, and elucidating what drives perceptions of stress in parents requires examining these factors in concert.

In parents, perceptions of control, loneliness, and childhood trauma have all been implicated in both parent and child outcomes. Perceived control in parents has been related to more positive parenting behaviors^58^ and locus of control has been linked to children’s self-regulatory behaviors^59,60^. Loneliness is associated with increased risk of abusive behaviors in parents^5^, indicating a potential role for loneliness in parents’ behavior and possibly responses to stress. Social support, a related, but independent, construct^61^, has also been linked to parenting behaviors, with higher levels of social support being associated with decreased negative parenting behaviors^62,63^. Additionally, parents with exposure to childhood trauma represent a particularly vulnerable group with increased risk for high levels of stress^45,64^ which in turn, has been linked to negative and less sensitive parenting behaviors^65^. Parasympathetic cardiac functioning has been associated with variability in sensitive parenting behaviors^66^ and increased emotion dysregulation during play interactions^67^. However, despite the recognition that perceptions of stress, control, childhood trauma, and, to a growing extent, loneliness and physiological functioning, are important to effective parenting and healthy child-parent relationships, these processes are rarely evaluated in concert^68^.

In the current research, we leverage data from two parallel studies conducted between 2015 and 2017 to examine the relationships between perceptions of control, loneliness, childhood trauma, and stress in parents. First, we conducted a cross-sectional study in primary caregivers examining how perceptions of control, loneliness, and childhood trauma experiences, along with socioeconomic factors and physiological indices linked to variability in stress perceptions, are related to caregivers’ reported stress. Next, we conducted a longitudinal study in a second sample of primary caregivers in which we examined how perceptions of control, loneliness, and childhood trauma experiences contribute to caregivers’ perceptions of stress. Additionally, we examined how perceptions of control, loneliness, and stress change over time, and whether changes in perceptions of control and loneliness, along with childhood trauma, are associated with changes in caregivers’ reported stress. Across these two studies we find robust evidence for relationships between caregivers’ perceptions of control, loneliness, and childhood trauma experiences and caregivers’ reported stress.

## Study 1

In Study 1, we examined how perceptions of control, loneliness, and childhood trauma exposure alongside indicators of socioeconomic strain contribute to perceived stress in 153 primary caregivers of children between the ages of 0–6 years old living in Oakland, CA. We expected that loneliness and childhood trauma would be associated with increased reported stress and perceptions of control would be associated with reduced reported stress. Additionally, we expected the effects of perceptions of control, loneliness, and childhood trauma on caregivers’ stress would be greater than those of indices of socioeconomic strain.

A secondary goal of this study was to examine whether parasympathetic autonomic cardiac functioning interacts with perceived control, loneliness, or prior experiences with stress to influence parents’ perceptions of stress. We expected that parents with higher resting PNS would demonstrate decreased perceptions of stress and that PNS would moderate the effects of control, loneliness, and prior exposure to stress on parents’ perceptions of stress.

### Method

#### Participants

Participants included 153 primary caregivers (29 male, 124 female) with at least one child between 0 and 6 years old who had not yet started kindergarten. Of these caregivers 16.3% were White, 21.6% White-Hispanic, 0.7% Hispanic, 24.2% Black, 12.4% Asian, 0.7% Hawaii Native/Pacific Islander, 21.6% Multi-Racial, and 2.6% Other. The mean age of caregivers was 34.6 years (SD = 7.45; 15–60 years) and average household income was $57,141.25 (SD = $51,102.60). Caregivers were sampled from five regions within Oakland, CA and included 17 total elementary school attendance areas defined by the Oakland Unified School District’s elementary school boundaries. This sample over-represented individuals of White-Hispanic race and ethnicity and was lower income as compared to the demographics of the broader United States determined by the most recent US Census data^69^. For additional recruitment details see Supplemental Materials.

#### Procedure

The current study was part of a broader study aimed at examining need for services in low income parents conducted in 2015. For the broader study, primarily in-home interviews were conducted with a primary caregiver(s) of the child(ren) of interest. Each interview lasted one-and-a-half to 2 h. During these interviews, participants were asked a range of questions about their own, their child’s, and the household’s demographics, access to services and health care, beliefs about parenting, household and neighborhood context, along with questions about primary caregivers’ affective processes. These questionnaires regarding affective processes were the primary variables of interest in the current study. Participants were provided with $50 in cash along with small toy prizes as incentives for participation. All participants provided written informed consent, and this study was approved by NORC at the University of Chicago’s Institutional Review Board and performed in accordance with relevant guidelines and regulations.

#### Primary measures of interest

Primary caregivers completed four questionnaires assessing perceived stress (*Perceived Stress Scale*; Cohen et al.^70^), loneliness (*UCLA Loneliness Scale-3*; Hughes et al.^71^), perceived control (*Pearlin’s Scale of Personal Mastery*; Pearlin and Schooler^72^; Eklund^73^), and childhood trauma experiences (*Adverse Childhood Experiences Scale; ACES*; Dube et al.^74^, Felitti et al.^75^). All scales are widely used and demonstrate good reliability and convergent validity. Internal reliability was calculated for each questionnaire using Cronbach’s alpha to determine each scale’s usefulness in the current sample. All questionnaires demonstrated good internal reliability (αs > 0.74).

#### Physiological measures

A subset of participants (n = 56) with complete data on the survey measures provided informed consent to wearing an ambulatory Zephyr Bioharness™ during the interview which was used to collect an electrocardiogram (ECG). Cardiovascular measures of parasympathetic cardiac control (high frequency heart rate variability; HF-HRV) and heart rate were derived from the ECG. ECG was collected at 250 Hz. MindWare software was used to visually inspect all ECG data. High frequency heart rate variability, a rhythmic fluctuation of heart rate in the respiratory frequency band that has been demonstrated to be a relatively pure index of parasympathetic cardiac control^76^, was derived from the ECG using spectral analysis of the interbeat interval series. The interbeat interval series was time sampled at 4 Hz (with interpolation) to yield an equal interval time series. This time series was detrended (second-order polynomial), end tapered, and submitted to a fast Fourier transformation. HF-HRV spectral power was then integrated over the respiratory frequency band (0.12–0.40 Hz) and is represented as respiratory sinus arrythmia (RSA), by taking the natural log of the heart period variance in the respiratory band (in ms^2^). Heart rate is reported in beats per minute. RSA and heart rate were coded for a ten minute period thirty minutes into the interview. This time period was chosen to allow time for participants to acclimate to the Zephyr Bioharness™ and interview context. RSA values ranged from 1.89 to 8.21 (M = 5.89, SD = 1.20) and heart rate ranged from 56.80 to 94.81 (M = 76.20, SD = 9.76).

#### Statistical analysis

To account for any variability associated with differences based on neighborhood and Oakland region, all analyses were run using three-level hierarchical linear models (HLM) with subject nested within elementary school attendance area nested within Oakland region in the lme4 package in R v.3.6.1^77^. For all models, predictor variables were continuous and standardized to reduce issues associated with collinearity and scaling^78^. Variance inflation factors (VIFs), which characterize how much the variance of the estimated coefficients increases due to collinear independent variables were calculated to confirm there were not concerns with collinearity. All VIFs were less than 5 which is generally considered acceptable^79^.

### Results

#### Effects of loneliness, perceived control, and childhood trauma experiences on perceived stress

**Figure 1:**
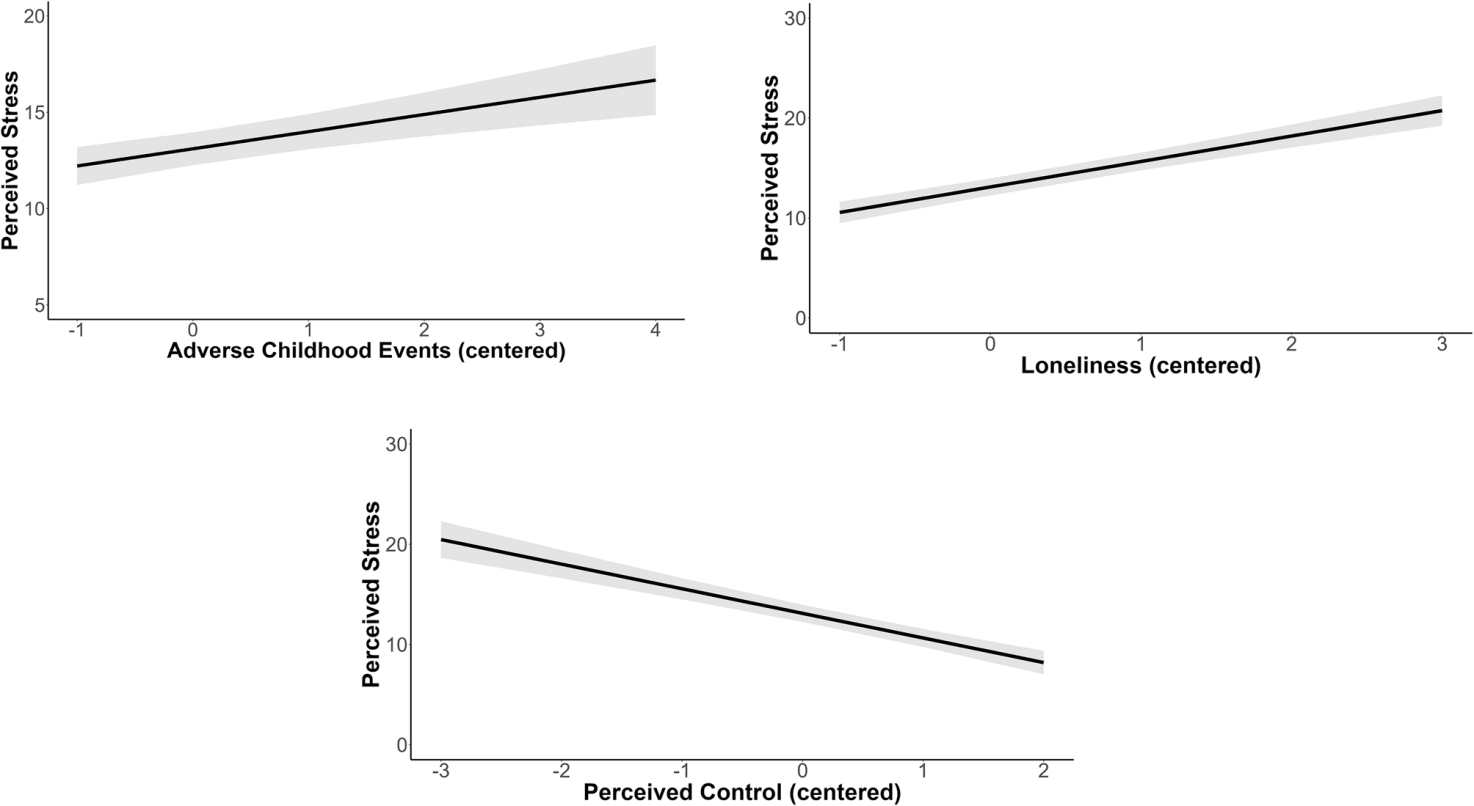
Effects of early childhood stress, loneliness, and perceived control on parents’ perceived stress. Gray bands represent confidence intervals.

We first assessed the effects of primary caregivers’ perceptions of loneliness, control, and childhood trauma (total score from 0 to 10 created by summing those items caregivers marked as experienced on the ACES) on perceived stress, controlling for income, race, gender, and age. As expected, loneliness (β = 2.55, SE = 0.44, *p* < 0.001) and childhood trauma (β = 0.89, SE = 0.40, *p* = 0.03) were both positively associated with caregivers’ perceived stress. Caregivers with higher levels of loneliness and individuals with greater exposure to childhood trauma had higher levels of current perceived stress (Fig. 1). Additionally, perceived control was negatively associated with perceived stress (β = − 2.45, SE = 0.46, *p* < 0.001), with individuals who had higher levels of perceived control having lower levels of perceived stress (Fig. 1). There were no significant effects for any of the control covariates (age, gender, race, income, and education) (ps > 0.05). Very little of the variance in the model was accounted for at the local elementary school area and region levels (< 0.0001), indicating that most of the model variance could be attributed to subject level factors. To assess the amount of variance accounted for by the fixed effects in the model (perceived loneliness, perceived control, stress, and gender, age, income, education, and race), the R^2^ was calculated using the methods outlined in Nakagawa and Schielzeth^80^). R^2^ = 0.49, indicating that this model accounts for roughly 49% of variance in subject level variation in perceived stress. Excluding income and education resulted in a one point change in explained variance (R^2^ = 0.48), suggesting that indicators of socioeconomic status do not account for a large amount of the explained variance in caregivers’ perceived stress.

#### Effects of resting parasympathetic activity on perceived stress

To confirm there were no systematic differences between the sample with complete questionnaire data and the sample with physiological indices (n = 56), we ran t-tests examining whether there were any significant differences in all outcome and predictor variables as well as demographic variables between participants with physiological data and those without. There was no evidence for differences in the two samples (ps > 0.10). To examine whether resting parasympathetic activity interacts with perceptions of control, loneliness, and early childhood trauma to influence caregiver perceptions of stress, we ran a model including interactions between RSA, perceived control, loneliness, and childhood stress (using the total ACES score). Given concerns that resting effects of parasympathetic activity may be accounted for by heart rate^81^, we also included heart rate and any interactions heart rate, perceived control, loneliness, and childhood stress in the model. There continued to be a negative relation between perceived control (β = − 3.58, SE = 0.68, *p* < 0.001) and a positive relation between loneliness (β = 2.18, SE = 0.69, *p* = 0.002) and perceived stress. However, the effect of childhood stress history was no longer significant (β = − 0.03, SE = 0.58, *p* = 0.96). There was a main effect of RSA on perceived stress, such that higher levels of resting parasympathetic activity were associated with higher levels of perceived stress (β = 1.61, SE = 0.79, *p* = 0.04).

**Figure 2:**
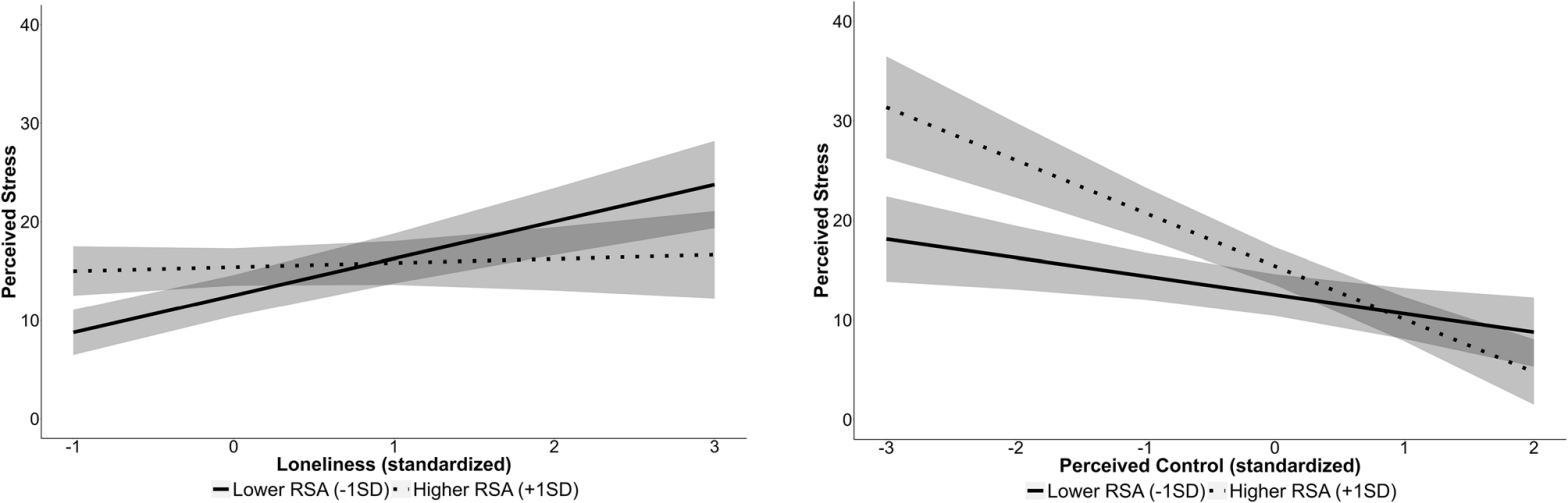
Interactions between PNS activity (RSA) and loneliness, and PNS activity (RSA) and perceived control on parents’ perceived stress. Here, simple slopes for RSA are plotted at one standard deviation above (higher RSA) and below (lower RSA) the mean as recommended by^100^. Gray bands represent confidence intervals.

As expected, there were significant interactions between RSA and perceived control (β = − 1.72, SE = 0.83, *p* = 0.04) and loneliness (β = − 1.75, SE = 0.82, p = 0.03; Fig. 2). Examining the simple slopes of these interactions at ± 1 SD for RSA indicated that individuals with higher RSA demonstrated a negative association between perceived control and stress (β = − 5.30, SE = 1.52, *p* = 0.001), but those with lower RSA did not (β = -1.86, SE = 1.32, *p* = 0.16). For loneliness, individuals with higher RSA demonstrated no association between loneliness and perceived stress (β = 0.43, SE = 1.51, *p* = 0.78), but there was a positive association for individuals with lower RSA (β = 3.93, SE = 1.31, *p* = 0.004). The R^2^ = 0.63 suggesting that this model accounts for 63% of the variance in subject level variation in perceived stress. Removing income and education from the model resulted in a change of 2%, again suggesting income and education didn’t account for a large amount of the explained variance in caregivers’ perceived stress.

### Discussion

We examined the effects of perceived control, loneliness, childhood trauma, resting parasympathetic activity, and indicators of socioeconomic strain on perceptions of current stress in caregivers. This work replicates previous research implicating perceptions of control and loneliness, along with early experiences with stress, on current perceptions of stress^11,22,24,44,45^. Specifically, we find that perceptions of control, loneliness, and early stress account for more variance than indices of environmental sources of socioeconomic stress (income, neighborhood, and education). Incorporating an understanding of the role of perception and differences in early experiences when examining contributors to caregiver stress can potentially support the development of more effective, targeted interventions aimed at subsets of the population in high risk environments. In particular, interventions that focus on changing parents’ perceptions of control and loneliness, as well as how they construe early childhood experiences, in addition to intervening on factors like income or neighborhood context, may be especially effective when trying to alleviate stress within the family.

In the subset of participants with resting parasympathetic cardiac measures, we found an effect of resting PNS activity on perceptions of stress. Surprisingly, we found higher resting PNS activity was associated with increased perceptions of stress. This is in contrast to previous research^47^. It is possible this discrepancy is due to differences in the samples being assessed. Our sample consisted of caregivers living in a predominantly low income area of the US, while previous research has primarily been conducted in college-aged students. Consistent with previous research^55^, resting PNS activity did moderate the relationship between perceptions of control and loneliness and perceived stress. High PNS was associated with a stronger negative relationship between perceived control and stress and a less positive relationship between loneliness and perceptions of stress. This suggests that individuals with high resting parasympathetic activity may be more susceptible to the stress dampening effects of high levels of perceived control and buffered from stress enhancing effects of loneliness. PNS activity is thought to index activity in neural circuits critical to flexible and adaptive responding^82,83^. Our findings are in line with this and suggest that the relationship between PNS and perceptions of stress may depend on individuals’ concurrent psychological states. However, these effects are preliminary and indicate a need for more research examining the relationship between PNS activity and perceived stress in different populations.

There are several limitations of the current research. The sample is from one location of the United States and predominantly White-Hispanic and low-income limiting the generalizability of the findings. Additionally, this research was cross-sectional making it difficult to determine the directionality of the observed relationships. The focus of the current research was to illuminate what factors influence individual differences in perceptions of stress, but it is likely that the relationships between perceived stress, control, loneliness, and parasympathetic nervous system functioning are bidirectional. In Study 2, we attempted to address some of these limitations, examining the relationships between perceived control, loneliness, childhood trauma, and stress and how these processes change over time in a second population of parents.

## Study 2

In our second study, we expanded on Study 1 in two primary ways. First, we sought to replicate our initial findings by examining the relationship between perceptions of control, loneliness, childhood trauma, and perceived stress in a second set of 154 primary caregivers of children ages 0–12 years living in Indiana. Second, we examined how these processes change over time. Specifically, we assessed whether caregivers’ perceptions of control, loneliness, and stress changed over the course of a 10-week period, and whether childhood trauma and changes in perceived control and loneliness relate to changes perceptions of stress. Based on our findings in Study 1, we expected perceived control to be negatively related to caregivers’ perceptions of stress and loneliness and childhood trauma to be positively related to perceptions of stress. We also expected perceptions of control, loneliness, and stress who change over the course of the 10 week period, and that changes in perceived control and loneliness, along with experiences of childhood trauma, would be related to changes in caregivers’ perceptions of stress.

### Method

#### Participants

All data were collected in collaboration with the Cooperative Family Development course provided by the Child And Parent Services (CAPS) non-profit organization in Elkhart, Indiana. This course is a 10-week-long stand-alone course that meets for 3 h once per week. It was created by CAPS for parents in the community and is based on the Systematic Training for Effective Parenting (STEP) curriculum^84^. Full details on the program can be found in the Supplemental Materials. Participants included 154 adults (92 female) who participated in the 10-week-long parenting course (between 2015 and 2017) and provided informed consent to participation in the current research study. Of these participants, 71.4% were White, 10.4% Hispanic, 11% African American, 3.2% Bi-Racial, and 3.9% other or non-disclosed. The mean age of participants was 30.68 years (SD = 8.63, 18–61 years; average number of children: 2, SD = 1.27; average age of children: 4.29 years, SD = 2.72). This sample is comparable to the demographics of the broader United States determined by the most recent US Census data^69^. As the study was implemented as an optional part of the parenting course curriculum, parents were not compensated for their participation. This study was approved by the University of Chicago Institutional Review Board and performed in accordance with relevant guidelines and regulations.

#### Survey measures

Participants completed the same survey measures assessing perceived stress, control, and loneliness as collected in Study 1 during week one and week ten of the parenting program. Parents also completed the *Childhood Trauma Questionnaire (CTQ)*^85^, a 28-item scale assessing parents’ exposure to trauma in childhood, specifically abuse and neglect, during week one. All questionnaires demonstrated good internal reliability (αs > 0.59). While income was not collected for all parents, median household income at a zip code level for each parent was calculated using the US Census Data from 2015. For questionnaire means and range of scores see Table S1.

#### Statistical analyses

Of the original 154 parents, 119 completed the course. Of these, 81 had complete data at both the beginning and the end of the 10 weeks. There were no differences in the outcome variables of interest at the start of the program for parents who had complete data at both time points (ps > 0.05). However, there was a significant difference in CTQ scores, such that parents who had complete data had lower CTQ scores than those who did not (t(118) =−2.28; *p* = 0.03). To examine relationships between perceived control, loneliness, childhood trauma, and stress at the beginning and end of the 10 weeks, linear regressions for each time point with stress as the outcome variable were run using the linear regression procedure in SPSS v24©^86^. All predictor variables were mean centered to reduce any issues related to collinearity^78^. To examine whether there were changes during the 10 weeks for perceived stress, loneliness, and perceived control, we ran one-way repeated measures ANOVAs including time (0 weeks and 10 weeks) as a within subjects factor using the general linear model repeated measures procedure in SPSS v.24©^86^. To examine how history of childhood trauma and changes in loneliness and perceived control relate to changes in perceived stress, a repeated measures ANOVA with perceived stress as the outcome variable, time as a within subjects factor, and total CTQ score and difference scores (calculated as post-pre) for control and loneliness as continuous covariates were run. The ANOVA was run as a full factorial including the cross products between CTQ score, change in loneliness, and change in control and time. To explore the directionality of any significant interaction effects, we used the MEMORE macro v 2.1^87^ which implements the methods for repeated measures moderation outlined in^88^). For all analyses, effect sizes are reported using partial η^2^ which represents the proportion of the effect plus error variance that is attributable to the effect and is recommended when reporting effect sizes for within subjects analyses^89^. Cohen^90^’s benchmarks to interpret η^2^ are η^2^ = 0.01 for small, η^2^ = 0.06 for medium, and η^2^ = 0.14 for large effect sizes. All models were run including age, gender, race, and income (median household income based on zip for entire sample) as covariates.

### Results

#### Relationship between childhood trauma, control, loneliness, and perceived stress

Regressions with perceived stress as the outcome variable and perceived control, loneliness, and childhood trauma as predictors indicated that perceived control (β = − 4.16, B = − 0.27, SE = 1.38, *p* = 0.003), loneliness (β = 1.09, B = 0.28, SE = 0.37, *p* = 0.004), and childhood trauma (β = 0.08, B = 0.22, SE = 0.03, *p* = 0.02) were all significantly associated with perceived stress. As observed in Study 1, higher loneliness and higher reported childhood trauma were associated with higher perceived stress, and higher perceived control was associated with decreased levels of perceived stress. A similar pattern was found for post program ratings with higher loneliness being associated with higher perceived stress (β = 1.18, B = 0.28, SE = 0.43 *p* = 0.008) and higher control being associated with lower perceived stress (β = − 5.18, B = − 0.44, SE = 1.20, *p* < 0.001). However, childhood trauma was no longer significantly associated with perceived stress (β = 0.01, B = 0.04, SE = 0.03, *p* = 0.71). There were no effects of age, race, gender, or income (ps > 0.05).

#### Changes in perceived stress, loneliness, and control

**Figure 3:**
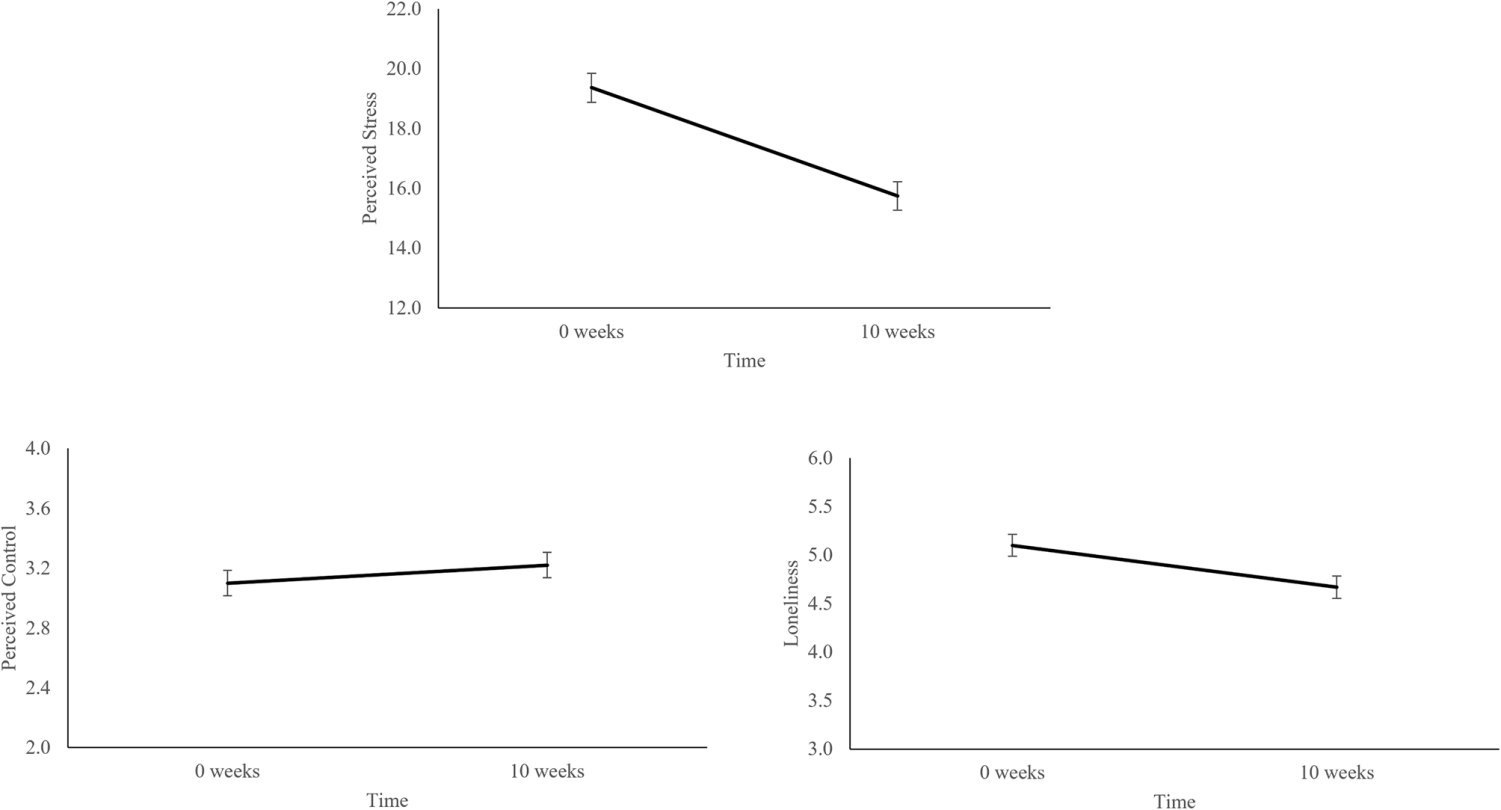
Change in perceived control, stress, and loneliness. Parents exhibited significant decreases in perceived stress and perceived loneliness. Significant increases were also observed in perceived control after including childhood trauma as a covariate. Error bars represent within subjects standard error calculated using methods outlined in^101^.

Repeated measures ANOVAs revealed that parents demonstrated significant change in perceived stress, loneliness, and perceived control between time point one and time point two. Parents showed significant decreases in perceived stress (F(1,102) = 24.23 *p* < 0.001, η^2^ = 0.19) and loneliness (F(1,90) = 4.80, *p* = 0.03, η^2^ = 0.05) and increases in perceived control (F(1,104) = 5.22, *p* = 0.02, η^2^ = 0.05) (Fig. 3) from the beginning to end of the 10 weeks. There were no effects of age, gender, or income (ps > 0.05). There was an interaction between race and time for changes in perceived control (F(1,104) = 8.06, *p* = 0.005, η^2^ = 0.07), such that the effect of time on perceived control was only significant for White individuals (β = 0.19, SE = 0.05, *p* < 0.001).

#### Relationships between childhood trauma, changes in control and loneliness, and perceived stress

**Figure 4:**
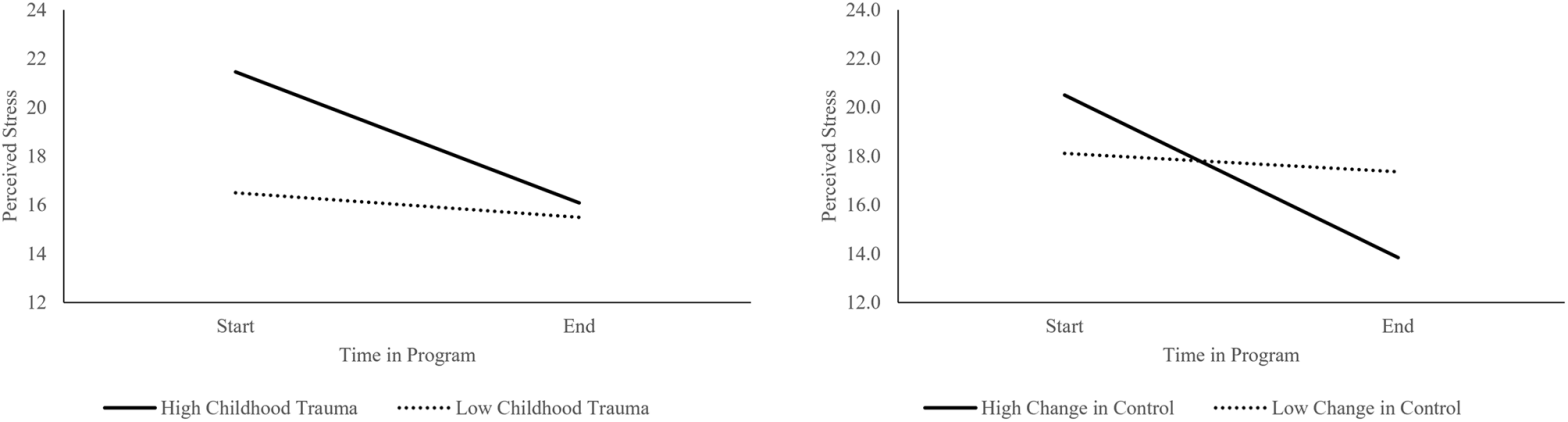
Effects of childhood trauma and changes in perceived control on changes in perceived stress overtime. Parents with higher childhood trauma scores and greater increases in perceived control over time demonstrated the greatest decreases in stress from the start to end of the program. Here, simple slopes are plotted at one standard deviation above and below the mean as recommended by^100^.

The main effect of time on perceived stress (F(1,73) = 25.34, *p* < 0.001, η^2^ = 0.26) remained significant after incorporating early childhood trauma, change in loneliness, and change in control as covariates. There was a significant interaction between change in perceptions of control and time (F(1,73) = 8.52, *p* = 0.005, η^2^ = 0.10). Moderation analyses indicated this was driven by greater increases in control being associated with larger decreases in stress over the 10 weeks (β = − 6.66, SE = 1.45, *p* < 0.001) (Fig. 4). There was also a significant interaction between childhood trauma and time (F(1,73) = 6.35, *p* = 0.01, η^2^ = 0.08). Moderation analyses indicated this was a result of higher childhood trauma exposure being associated with larger decreases in perceived stress over the 10 weeks (β = − 0.10, SE = 0.03, *p* = 0.003, Fig. 4). There were no significant effects of changes in loneliness on perceived stress (F(1,73) = 1.02, *p* = 0.32, η^2^ = 0.01). Together this suggests children with higher childhood trauma demonstrated more pronounced decreases in stress over the course of the program and individuals who demonstrated the most pronounced increases in control demonstrated the largest decreases in stress. There were no effects of age, race, gender or income (ps > 0.10).

### Discussion

In Study 2, we replicated our previous results in a second sample of parents, finding higher levels of perceived control were associated with decreased levels of stress, and higher levels of loneliness and childhood trauma were associated with increased perceived stress. This suggests that the relationships between control, stress, loneliness, and childhood trauma are stable across different populations of parents. Additionally, we found that over the course of 10 weeks parents’ perceptions of stress and loneliness decreased while those of control increased and changes in control along with childhood trauma were related to changes in stress. Specifically, higher childhood trauma history and larger increases in control were associated with more pronounced decreases in stress.

These findings are consistent with changes in stress over time being linked to changes in perceptions of control. This is in line with the wealth of research implicating perceptions of control as a critical factor in shaping perceptions of stress and stress responses^24,25,91^, and with research implicating perceptions of control in stress related disorders and psychopathology like post-traumatic stress disorder (PTSD)^92–94^. However, it indicates an area of potential intervention for parents experiencing high levels of stress—providing parents with tools aimed at helping them better appreciate and effectively use aspects of control they have over both parenting and more generally has the potential to decrease alleviate levels of stress. Indeed, research with older adults finds that small interventions which shift perceptions of control, such as providing individuals with a plant to care for, decreases reported stress and increases reported well-being, as well as improves health outcomes^95–97^.

We also find childhood trauma history is associated with changes in stress, particularly larger decreases in stress over the course of the 10 weeks. This is somewhat surprising given childhood trauma has been associated with increased perceptions of stress^98^, as well as increased loneliness and social isolation^99^. This would suggest parents with higher reported childhood trauma would be more likely to demonstrate chronic high levels of stress. However, this effect may be a result of the fact that all caregivers were also participating in a parenting program congruent to data collection. It is possible that caregivers with high levels of childhood trauma were most susceptible to any stress reducing effects of the program. However, we cannot draw conclusions about the program itself in the absence of a control group. Future research aimed at intervention assessment should examine these questions in the context of a randomized control trial. Last, our study was not able to determine to what extent the changes in stress, loneliness, and control are maintained beyond the 10 weeks of the parenting program. More research assessing perceptions of control, stress, and loneliness across a longer period of time is necessary to determine the stability of these patterns.

## Overall discussion

In this research, we examined the effects of perceived control, loneliness, and childhood trauma experiences on perceptions of stress in two populations of caregivers with data collected between 2015 and 2017. In both populations, we found robust evidence implicating perceptions of control and loneliness, along with childhood trauma experiences, in current perceptions of stress. Specifically, consistent with previous research, we found that higher levels of perceived control were associated with decreased levels of stress, and higher levels of loneliness and childhood trauma were associated with increased perceived stress. Additionally, we find changes in control and childhood trauma were related to changes in stress, such that higher childhood trauma history and larger increases in control were associated with more pronounced decreases in stress.

These findings suggest that history of childhood trauma, loneliness, and control all independently contribute to parents’ perceptions of stress. Additionally, our findings in Study 1 indicate that these factors account for more variance in perceptions of stress than indices of environmental sources of socioeconomic stress. These relationships are in line theoretical models of stress^9–12^, which posit control, social relationships, and early experience as critical to shaping perceptions of stress. They suggest it is important to account for both perceptual and environmental factors when examining what contributes to variability in parents’ stress. Our finding in Study 2 that stress reductions were most pronounced in parents who demonstrated greater increases in control indicate that interventions aimed at increasing feelings of control may be particularly effective. These findings also indicate that interventions targeting parents’ perceptions of stress, along with loneliness and control, may demonstrate greater efficacy for parents with prior experiences of trauma, a population considered at greater risk for stress later in life.

There are several limitations of the current research. Given this research assessed only parents’ outcomes, it is important that future research examines whether the changes in stress, loneliness and control in parents translate into positive developmental outcomes for their children. Additionally, while the parenting program in Study 2 provided a unique opportunity to examine within-individual changes in these processes, it somewhat limits the generalizability of these effects outside the context of the program, as changes may be driven by program participation. We also were unable to collect individual level data on income and relied on census level data based on zip code, limiting any findings related to SES in this study. However, these data still provide insight into how childhood trauma, control, and loneliness relate to parents’ perceptions of stress and how changes in control and loneliness contribute to changes in stress over time. The current research represents a small but potentially important initial step in a larger effort to address whether these programs are associated with positive changes for individuals at high risk of negative outcomes associated with parenting and if these changes differ from other groups of parents. The samples across the two studies also differed slightly in terms of age and race and ethnicity, which may limit comparability. But the fact we do find comparable results across the two samples suggests the effects are robust. Last, the current work did not assess all potential factors that could contribute to variation in individuals’ perceptions of stress. Future should work examine how other factors such as depression, anxiety, and perceived predictability and safety contribute to parents’ perceptions of stress.

Despite these limitations, these studies provide insight into the joint contributions of perceived control, loneliness, and childhood trauma to perceptions of stress in parents. This research suggests that parents’ stress related processes are malleable and has the potential to aid in informing interventions aimed at reducing high levels of stress within this population. Future work should aim to identify types of activities and interventions are effective at changing perceptions of control, and through this stress. Additionally, this research suggests there are important questions involving the relationships between parents’ perceptions of their environment and more “objective” risk factors, such as income, and their role in parents’ perceptions of stress. Better understanding these relationships could help inform our understanding of which aspects may be the most effective focus for intervention and represents an exciting opportunity towards furthering understanding of family dynamics and functioning.

### Supplementary Information

Supplementary Information.

## Data Availability

All associated data and code is available on the Open Science Framework (OSF; https://osf.io/9qt7s/).
