# Supplementary material for: Perceived control, loneliness, early-life stress, and parents’ perceptions of stress
Source: Sci Rep. 2023 Aug 10;13:13037. doi: 10.1038/s41598-023-39572-x (PMC10415274; doi:10.1038/s41598-023-39572-x)
Supplement: Supplementary file 1 — Supplementary Information. [file 41598_2023_39572_MOESM1_ESM.docx]

**Perceived control, loneliness, early-life stress, and parents’ perceptions of stress: Convergent evidence from two populations**

Karen E. Smith, Eileen Graf, Kelly E. Faig, Stephanie J. Dimitroff, Frederica Rockwood, Marc W. Hernandez, Greg J. Norman

Supplemental Materials

Study 1

Additional Recruitment Details

Families were not sampled from school classrooms but rather the geographic areas that form each school attendance area. This sampling procedure was used to ensure a sample geographically representative of the broader Oakland area, and reduce any potential sample bias towards certain Oakland areas. Initially 7,905 households in Oakland were sampled randomly, from which 560 eligible households were identified. Of these 560 eligible households, 426 completed the interview. Participants were given the option to not respond to all interview questions, and because of this complete data on the primary measures of interest was only available for 153 of the total 426 participants.

Additional Questionnaire Details

**Perceived stress.** The *Perceived Stress Scale* ^1^, a 10-item scale assessing individuals’ perceptions of stress in the past month using a Likert scale ranging from 0 to 4, was used to assess caregivers’ current global perceptions of stress. This scale includes items such as “In the last month, how often have you felt stressed?” and “In the last month, how often have you felt difficulties piling up so high that you could not control them”. Perceived stress scores ranged from 1 – 32 (M = 13.93, SD = 6.35).

**Loneliness.** To assess caregivers’ loneliness, the *Three-Item Loneliness Scale* ^2^, a 3-item scale assessing individuals’ perceptions of loneliness using a Likert scale ranging from 1 to 3, was used. This scale was designed specifically for measuring loneliness in large surveys, as in the case of the current study, and is based on the 20-item Revised UCLA Loneliness scale ^3^. The three-item scale has demonstrated similar results to the full scale ^2^ and includes items such as “How often do you feel isolated from others?”. Loneliness scores ranged from 3 – 9 (M = 4.57, SD = 1.65)

**Perceived control.** *Pearlin’s Scale of Personal Mastery* ^4,5^, a 7-item scale assessing individuals’ perceptions of control in their lives using a Likert scale ranging from 1 to 4, was used to assess caregivers’ perceptions of control. Items include questions such as “I have little control over the things that happen to me” and “There is little I can do to change many of the important things in my life”. Perceived control scores ranged from 1.71 – 4 (M = 3.20, SD = .53)

**Early childhood stress.** To assess childhood experiences with stress, the *Adverse Childhood Experiences Scale (ACES)* ^6,7^, a 10-item scale asking individuals whether or not they experienced 10 different early adverse events prior to 18 years of age, was used. ACES ranged from 0 – 9 (M = 1.84, SD = 2.06).

Additional Analytic Details

As there was missing data due to participants having the option to not respond, we examined whether there were any significant differences in all outcome and predictor variables as well as demographic variables between participants with complete data and those with incomplete data. Participants without complete data were not significantly different from those with complete data on any of the primary variables of interest (perceived stress, perceived control, loneliness, and childhood stress) (p > 0.05). However, the sample with complete data was significantly older (34.6 years) than the sample without (32.8; t_322.17_ = 2.32, p = 0.020) and reported a higher level of income (Complete: $57,141.25, Incomplete: $44,071.78; t_252.73_ = 2.16, p = 0.032) and education (Complete: 4.21, Incomplete: 3.02; t_291.86_ = 6.57, p < 0.001). Additionally, there were differences in the racial composition of the sample with complete data compared to the sample without (Table S2). Given participants did not differ on the primary outcomes and predictors of interest, we determined data was missing at random and opted to use listwise deletion rather than data imputation measures ^8^. As participants were initially sampled based on elementary school attendance area within each Oakland region, and hierarchical linear models are particularly suited to handling this type of data ^9^, a three-level hierarchical linear model (HLM), with subject nested within elementary school attendance area nested within Oakland region.

HLM techniques are a complex form of ordinary least square (OLS) regression which fits a linear function to the observed data while accounting for variation across individuals to estimate the population level relationship based on the observed data set ^8,9^. These models are preferable over an analysis of variance approach for data that are nested, and HLM accounts for potential variation in observed outcomes across individuals ^8,9^. Given the participant racial distribution, where White Non-Hispanic, the typical comparison group for analyses of race, was only a small proportion of the total sample (16.3%), we instead grouped race as Hispanic-White (21.6%), Multi-Racial (21.6%), Black (24.2%), and Other (consisting of individual who reported their race as either: White Non-Hispanic (16.3%), Hispanic (0.7%), Asian 12.4%), Hawaii Native/Pacific Islander (0.7%), and Other (2.6%)) and dummy coded such that the comparison group was White-Hispanic.

Additional Results

Effects of Heart Rate

There were also significant interactions between heart rate and perceived control (β = -2.60, SE = 0.70, p < 0.001), loneliness (β = -1.57, SE = 0.76, p = 0.04), and childhood stress history (β = 1.69, SE = 0.80, p = 0.04). For loneliness and perceived control, examining the simple slopes for ±1 SD for heart rate demonstrated a similar pattern to that of RSA. For childhood stress, the interaction appeared to be driven by children with lower heart rate demonstrating a negative association between childhood stress history and perceived stress while those with higher demonstrated a positive association.

Study 2

Cooperative Family Development Course

The Cooperative Family Development course is a ten-week-long stand-alone course that meets for three hours once per week provided and created by CAPS for parents in the community. The course is based on the Systematic Training for Effective Parenting (STEP) curriculum ^10^. Trained instructors employed by CAPS lead the courses which are offered throughout the year with the goal of teaching parents positive parenting tools to help them raise children who are happy, healthy, compassionate, cooperative, and responsible. They include parent-child activities, one-on-one coaching for parents, and in-class learning and support. Specifically, these classes aim to promote positive parenting behaviors, reduce power struggles, encourage responsibility on the part of the parent, teach parents how to apply consequences to their child in a manner that teaches and helps the child, and control family stress.

The Cooperative Family Development course consists of group classes during which parents are taught through instruction and videos. Parents are also given handouts in which they are asked to work through a variety of possible parenting scenarios using the tools they have been taught. The class consists of eight different topics related to parenting: 1) parenting styles; 2) child development and learning through play; 3) making positive child directed statements and avoiding giving into child demands; 4) understanding why children misbehave; 5) implementing natural and logical consequences; 6) understanding your child’s feelings and the importance of expressing those feelings in a helpful manner; 7) managing stress; and 8) problem solving and course review.

During the “parenting styles” module (based on materials from ^10–13^, parents discuss three types of parenting styles, identify which style they fall into and how their emotional state (i.e. feeling stressed) influences their parenting behaviors, and are provided with tools to encourage a democratic style of parenting that emphasizes giving choices.

The “child development and learning through play” module (based on materials from ^14–16^ concentrates on developing realistic expectations of child development that allow parents to encourage and empower their child toward self-discipline. This module includes teaching an understanding of the concept that children learn through play, providing parents with tools to use play to nurture their child’s growth.

The “making positive child-directed statements and avoiding giving into child demands” module (based on materials from ^17–19^ involves discussion and practice of positive strategies for parents to employ when children exhibit difficult behaviors such as whining, acting angry, and pouting. The goal of this module is to teach parents to identify when their child is trying to manipulate them and how to respond to that behavior in a calm, positive, consistent manner.

In the “understanding why children misbehave” module (based on materials ^10,11,20^, parents are taught that children’s misbehavior can be driven by a variety of motivations, including hunger, illness, need for attention, anger etc. Parents are then provided with tools to better identify reasons for their own child’s misbehavior and strategies to respond in a constructive manner.

In the “implementing natural and logical consequences” module (based on materials from ^10,21,22^, parents are taught to practice positive strategies for disciplining their child as an alternative to punishment, focusing on how to effectively outline natural and logical consequences of a behavior to a child in order to instill self-discipline. As part of this module, parents are asked to plan and implement appropriate consequences based on scenarios of a child’s misbehavior.

“Understanding your child’s feelings and the importance of expressing those feelings” (based on materials from ^23–25^ focuses on teaching parents the importance of their role as caregiver in helping a child learn how to recognize and appropriately express their feelings, especially frustration or anger. It also teaches parents to be better aware of their own feelings and to identify healthy expression of those feelings so they can model this skill to their child.

The “managing stress” module (based on materials from ^26–28^ concentrates on developing an understanding in parents that stress is a necessary part of life and can motivate positive behaviors, but too much prolonged stress is damaging. Parents’ are taught methods for managing responses to life stressors and for creating a low stress environment for their child.

The last module, “problem solving and review” (based on materials from ^10,29^, focuses on learning strategies for effective problem solving with their spouse, children and family to help reduce stress and improve the family’s ability to function healthily. Additionally, this module incorporates a final activity in which parents are presented with a variety of scenarios and asked to apply the tools they’ve learned over the course of the class in their responses.

As the course is offered by CAPS to the general community, parents include individuals who are self-referred (53.9%), referred by the legal system (36.4%) (attorney, court, Department of Child Services, Department of Health Services, Guardian ad Litem, Probation Officer), or referred by other community organizations (7.8%) (Parent Aide, parenting class staff members, schools, churches, or Youth Services Bureau). Referral data was not available for 1.9% (3) participants. Of the 154 parents, 72 participated with a co-parent, 81 did not, and there was 1 participant for whom this information was not available.

Additional Analytic Details

Given data was collected in the context of the Cooperative Family Development Course across several classes which varied by teacher, and participants varied in why they were referred to the class and whether they took the class with a co-parent, we ran one way ANOVAs examining whether these variables were related to the primary outcomes of interest (perceived stress, loneliness, and perceived control). Only class teacher was related to perceived control both pre and post program participation (Pre: F = 2.70, p = 0.01; Post: F = 3.29, p = 0.003). All other relations were not significant (ps > 0.05). Given this relation between teacher and perceived control, we ran the model assessing changes in control over time including class teacher as a between subjects factor. This model did not change the reported effects.

Table S1

*Means and standard deviations of outcome measures before and after the parenting program for Study 2*

| Outcome | Mean Beginning of Parenting Program (SD) | Mean End of Parenting Program (SD) | Range of scores for scale |
| --- | --- | --- | --- |
| Control | 3.08  (0.46) | 3.22  (0.51) | 1 – 7 |
| Stress | 19.71  (7.07) | 15.57  (6.35) | 0 – 40 |
| Loneliness | 5.02  (1.90) | 4.66  (1.61) | 3 - 9 |
| Childhood Trauma Scale | 48.28  (22.22) | - | 25 - 140 |

Table S2

*Comparisons of racial composition in participants with complete data and participants without complete data for Study 1*

| **Race** | **% Complete** | **% Incomplete** | **t-value** | **df** |
| --- | --- | --- | --- | --- |
| **White** | 16.3 | 4.0 | 3.81*** | 201.1 |
| **White-Hispanic** | 21.6 | 30.4 | -2.03* | 344.67 |
| **Hispanic** | 0.7 | 1.1 | -0.49 | 382.4 |
| **Black** | 24.2 | 14.7 | 2.34* | 268.2 |
| **Asian** | 12.4 | 6.2 | 2.03* | 244.6 |
| **Hawaii Native/Pacific Islander** | 0.7 | 0 | 1 | 152 |
| **Multi-Racial** | 21.6 | 31.1 | -2.19* | 346.45 |
| **Other** | 2.6 | 12.5 | -4.13*** | 416.6 |

*Note:* Significance levels *p<0.05, **p< 0.01, ***p<0.001

References

1. Cohen, S., Kamarck, T. & Mermelstein, R. A Global Measure of Perceived Stress. *Journal of Health and Social Behavior* **24**, 385–396 (1983).

2. Hughes, M. E., Waite, L. J., Hawkley, L. C. & Cacioppo, J. T. A Short Scale for Measuring Loneliness in Large Surveys: Results From Two Population-Based Studies. *Research on aging* **26**, 655–672 (2004).

3. Russell, D. W. UCLA Loneliness Scale (Version 3): Reliability, validity, and factor structure. *Journal of Personality Assessment* **66**, 20–40 (1996).

4. Pearlin, L. I. & Schooler, C. The structure of coping. *Journal of Health and Social Behavior* **19**, 2–21 (1978).

5. Eklund, M., Erlandsson, L.-K. & Hagell, P. Psychometric properties of a Swedish version of the Pearlin Mastery Scale in people with mental illness and healthy people. *Nordic Journal of Psychiatry* **66**, 380–388 (2012).

6. Felitti, V. J. *et al.* Relationship of childhood abuse and household dysfunction to many of the leading causes of death in adults. The Adverse Childhood Experiences (ACE) Study. *American Journal of Preventive Medicine* **14**, 245–258 (1998).

7. Dube, S. R., Williamson, D. F., Thompson, T., Felitti, V. J. & Anda, R. F. Assessing the reliability of retrospective reports of adverse childhood experiences among adult HMO members attending a primary care clinic. *Child Abuse and Neglect* **28**, 729–737 (2004).

8. Singer, J. D. & Willett, J. B. *Applied longitudinal data analysis: Modeling change and event occurrence*. (Oxford University Press, 2003).

9. Raudenbush, S. W. & Bryk, A. S. *Hierarchical linear models: Applications and data analysis methods*. (Sage Publications, 2002).

10. Dinkmeyer, D. & McKay, G. *The parent’s handbook: Systematic training for effective parenting*. (American Guidance Service, 1989).

11. *Winning at parenting: Without beating your kids*. (Kids are worth it!, 1989).

12. Popkin, M. H. Active Parenting: 30 years of video-based parenting educatoin. *The Journal of Individual Psychology* **70**, 166–175 (2014).

13. Lott, L. & Nelson, J. *Teaching parenting the positive discipline way*. (Sunrise, 1995).

14. Fahlberg, V. *Attachment and separation (Vol 429)*. (Michigan Department of Social Services, 1979).

15. Borba, M. *Building moral intelligence: The seven essential virtues that teach kids to do the right thing*. (Jessey-Bass, 2001).

16. Reiner, I. *et al.* Methylation of the oxytocin receptor gene in clinically depressed patients compared to controls: The role of OXTR rs53576 genotype. *Journal of Psychiatric Research* **65**, 9–15 (2015).

17. Shannon, D. *No David!* (Blue Sky Press, 1998).

18. Coloroso, B. *Kids are worth it!: [giving your child the gift of inner discipline]*. (Penguin Books, 1999).

19. Svabic, I. & Martin, P. *No & Yes: An active toddler’s guid on how to behave!* (Chicken House/Scholastic, 2002).

20. Kovls-Reidler, B. & Kvols-Reidler, K. *Redirecting children’s misbehavior*. (R.D.I.C. Publications, 1979).

21. Duffy, R., Erwin, C. & Nelson, J. *Positive discipline: The first three years, revised and updated edition: From infant to toddler - Laying the foundation for raising a capable, confident child*. (Harmony Publishing, 2015).

22. Faber, A. & Mazlish, E. *How to talk so kids will listen & listen so kids will talk*. (Rawson, Wade Publishers, 1980).

23. Davis, L. & Keyser, J. *Becoming the parent you want to be: A sourcebook of strategies for the first five years*. (Harmony Publishing, 1997).

24. 24/7 Dad. *© 2014 National Fatherhood Initiative* https://store.fatherhood.org/24-7-dad-am-3rd-ed-complete-program-kit.

25. Robberecht, T. & Goossens, P. *Angry dragon*. (Clarion Books, 2004).

26. Hardt, J. & Rutter, M. Validity of adult retrospective reports of adverse childhood experiences: Review of the evidence. *Journal of Child Psychology and Psychiatry and Allied Disciplines* **45**, 260–273 (2004).

27. Boyce, L. *When you work curriculum sourcebook*. (University of Wisconsin Extnesion, Cooperative Extension, 1997).

28. Holmes, T. & Rahe, R. The social readjustment rating scale. *Journal of Psychosomatic Research1* **11**, 213–218 (967).

29. *Therapeutic Crisis Intervention System: Information Bulletin*. (The Residential Child Care Project. Bronfenbrenner Center for Translational Research. College of Human Ecology, 2010).
